# Using mercury stable isotope fractionation to identify the contribution of historical mercury mining sources present in downstream water, sediment and fish

**DOI:** 10.3389/fenvc.2023.1096199

**Published:** 2023-02-09

**Authors:** Chris S. Eckley, Collin Eagles-Smith, Todd P. Luxton, Joel Hoffman, Sarah Janssen

**Affiliations:** 1 U.S. EPA, Region 10, Seattle, WA, United States,; 2 U.S. Geological Survey, Forest and Rangeland Ecosystem Science Center, Corvallis, OR, United States,; 3 US EPA ORD, Center for Environmental Solutions and Emergency Response, Cincinnati, OH, United States,; 4 U.S. EPA Office of Research and Development, Center for Computational Toxicology and Exposure, Great Lakes Toxicology and Ecology Division, Duluth, MN, United States,; 5 U.S. Geological Survey, Mercury Research Lab, Upper Midwest Water Science Center, Madison, WI, United States

**Keywords:** mercury, stable isotopes, contaminated sites, reservoirs, abandoned mines

## Abstract

Ecosystems downstream of mercury (Hg) contaminated sites can be impacted by both localized releases as well as Hg deposited to the watershed from atmospheric transport. Identifying the source of Hg in water, sediment, and fish downstream of contaminated sites is important for determining the effectiveness of source-control remediation actions. This study uses measurements of Hg stable isotopes in soil, sediment, water, and fish to differentiate between Hg from an abandoned Hg mine from non-mine-related sources. The study site is located within the Willamette River watershed (Oregon, United States), which includes free-flowing river segments and a reservoir downstream of the mine. The concentrations of total-Hg (THg) in the reservoir fish were 4-fold higher than those further downstream (>90 km) from the mine site in free-flowing sections of the river. Mercury stable isotope fractionation analysis showed that the mine tailings (δ^202^Hg: −0.36‰ ± 0.03‰) had a distinctive isotopic composition compared to background soils (δ^202^Hg: −2.30‰ ± 0.25‰). Similar differences in isotopic composition were observed between stream water that flowed through the tailings (particulate bound δ^202^Hg: −0.58‰; dissolved: −0.91‰) versus a background stream (particle-bound δ^202^Hg: −2.36‰; dissolved: −2.09‰). Within the reservoir sediment, the Hg isotopic composition indicated that the proportion of the Hg related to mine-release increased with THg concentrations. However, in the fish samples the opposite trend was observed—the degree of mine-related Hg was lower in fish with the higher THg concentrations. While sediment concentrations clearly show the influence of the mine, the relationship in fish is more complicated due to differences in methylmercury (MeHg) formation and the foraging behavior of different fish species. The fish tissue δ^13^C and Δ^199^Hg values indicate that there is a higher influence of mine-sourced Hg in fish feeding in a more sediment-based food web and less so in planktonic and littoral-based food webs. Identifying the relative proportion of Hg from local contaminated site can help inform remediation decisions, especially when the relationship between total Hg concentrations and sources do not show similar covariation between abiotic and biotic media.

## 1 Introduction

Mercury (Hg) is a pollutant of global concern due to its widespread atmospheric distribution and potential to impact remote areas (Obrist et al., 2018). Aquatic ecosystems exposed to Hg pollution are also often susceptible to the conversion of inorganic Hg to methylmercury (MeHg) by anaerobic microorganisms, which is modulated by Hg bioavailability, carbon sources, and terminal electron acceptor concentrations (e.g., sulfate, ferric iron) (Bravo and Cosio, 2020). After formation, MeHg is readily bioaccumulated in aquatic food webs, resulting in fish consumption advisories across the United States (US) and globally (Munthe et al., 2007). Mitigating Hg contamination and subsequent fish consumption advisories can be extremely difficult, particularly because Hg can enter the environment *via* numerous routes. In addition to inputs of atmospheric Hg, there are also local sources of Hg pollution that can contribute to Hg exposure and risk (Eckley et al., 2020). In the western US, there are hundreds of thousands of Hg contaminated sites that have contributed to elevated fish MeHg concentrations, often including defunct facilities such as abandoned mines and former industrial/manufacturing plants (Alpers et al., 2016; Eagles-Smith et al., 2016c).

Distinguishing the relative contributions of Hg from multiple sources that have accumulated in fish is complicated by differences in inorganic Hg bioavailability for methylation. The elevated Hg concentrations released from mining areas often have lower bioavailability than atmospheric sources (Suchanek et al., 2008; Eckley et al., 2017), though this is not the case for all types of contaminated sites (Janssen et al., 2021b). Uncertainties regarding the sources of MeHg that accumulates in fish obfuscates setting meaningful remediation goals at contaminated sites. Due to the unique biogeochemical conditions in a waterbody that govern how much MeHg is produced, it is very difficult to identify what “background” levels would be in the absence of releases from a local point source (Watras et al., 1995). As a result, a major challenge exists in determining the proportion of MeHg in fish that originated from local releases versus other sources such as atmospheric deposition. This is further complicated when the point of concern about elevated Hg in fish occurs many kilometers downstream (e.g., a reservoir or estuary) from the location of releases from a contaminated site (i.e., the abandoned mine or industry) (Park and Curtis, 1997; Foucher et al., 2009; Baptista-Salazar and Biester, 2019). As such, there remains large uncertainty regarding how far downstream from a contaminated site the fish Hg levels are impacted from a particular industrial source. Source attribution is further complicated by the multiple variables that influence the amount of Hg that accumulates in fish tissue, which includes the uptake and biomagnification of MeHg within the aquatic foodweb (Eagles-Smith et al., 2016a).

An approach used to help understand the cycling and sources of Hg in the environment involves the analysis of the fractionation of Hg stable isotopes (Blum et al., 2014; Yin et al., 2014). During kinetic and equilibrium reactions, Hg isotopes undergo mass dependent fractionation (MDF), which is commonly represented utilizing δ^202^Hg (Blum and Bergquist, 2007). Mass independent fractionation (MIF) can also occur due to magnetic isotope and nuclear volume effects (Bergquist and Blum, 2007; Zheng and Hintelmann, 2010a; Zheng and Hintelmann, 2010b) and is most commonly observed in the odd-isotopes (^199^Hg and 201Hg). Separate to these processes and commonly detected first in atmospheric media, MIF of multiple isotopes occurs but due to complicating factors arising from odd-isotope MIF, are reported as even-isotope MIF (^200^Hg and ^204^Hg) (Fu et al., 2021) but the precise mechanisms are not well agreed upon. While MDF is often used to trace sources and processes, MIF is commonly used to identify photochemical reactions in aquatic environments (Bergquist and Blum, 2007; Zheng and Hintelmann, 2010a).

This study was designed to assess the potential contributions of point source Hg to fish tissue downstream from a historical mining site, the Black Butte mine. To effectively resolve mixed Hg sources and bioaccumulation differences driven by habitat, we used an integrated approach of examining foodweb dynamics alongside abiotic fate and transport processes. We were specifically interested in testing the hypothesesis that fish in the river/reservoir system are impacted by releases of Hg sourced from Black Butte mine. This hypothesis was addressed by utilizing stable Hg isotope values in mine-site materials and downstream water, sediments, and fish tissue to assess the impacts of legacy contamination in the upper watershed on downstream fish concentrations. The holistic approach of examining multiple matrices for Hg stable isotopes will also provide valuable insight on how Hg is transformed through watershed transport away from contaminated point sources.

## 2 Materials and methods

### 2.1 Site description

**FIGURE 1:**
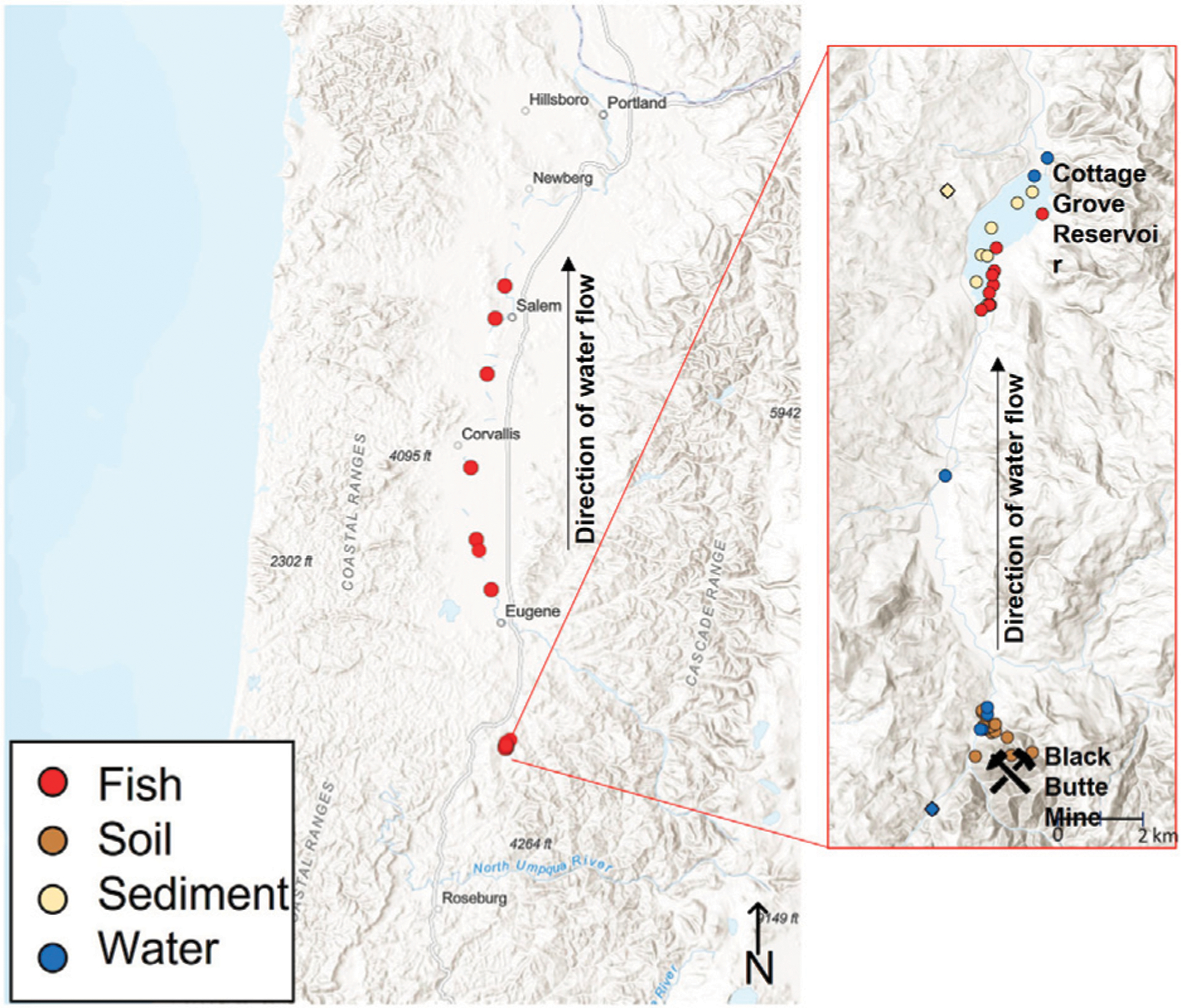
Map showing locations where samples were collected. Fish were collected over a larger geographic area than the abiotic parameters (soil, sediment, and water). Colors denote collection locations of different matrices. Circles represent locations downstream of the mine and diamonds represent background samples.

The study area focuses on Black Butte mine and its downstream waterbodies, which are located within Oregon, United States (Figure 1). Black Butte mine is a historical Hg mine and smelter that operated from the 1890s to the 1960s. The operation produced approximately 635,000 kg of Hg and over 200,000 m^3^ of waste. There are three general types of mine-waste present at the site: Tailings that were generated from an older furnace (operated until 1909), tailings generated from a newer furnace, and mine waste material that was not processed (e.g., waste rock that was removed during the mining process but did not contain sufficient Hg to be sent to a furnace).

Furnace Creek drains directly through the mine tailings and flows into Little River and then the Coast Fork Willamette River 2 km further downstream. At approximately 15 km downstream of Black Butte mine is the Cottage Grove Reservoir. This study focuses on the reservoir because it contains some of the highest fish tissue Hg concentrations within this region and is a popular fishing destination (Ambers and Hygelund, 2001; Curtis et al., 2013). Previous studies have focused on Hg methylation dynamics within the reservoir (Eckley et al., 2015; Eckley et al., 2017); however, that work did not address the relative proportion of Hg that originated from the legacy mine site. Sampling of fish extended beyond Cottage Grove Reservoir to identify if there are potential impacts on fish isotopic Hg compositions over 90 km downstream of the mine site in the Willamette River.

The water level of Cottage Grove Reservoir varies significantly throughout the year because it is managed to control flooding. At full pool conditions (24 m at its deepest during May to September), vegetated wetland areas along the edges of the reservoir become inundated with water, covering approximately 8% (~36 ha) of the total surface area of the reservoir during the summer months (Eckley et al., 2017). To have storage capacity to prevent flooding, the reservoir water level is lowered before the part of the year that experiences higher rainfall (September through April). During the low pool conditions, over 60% of the reservoir area is exposed sediment and the dried wetlands are not directly hydrologically connected to the reservoir through surface water. During dryer years, the reservoir may not have enough inflow discharge to reach or maintain full pool conditions. If the reservoir does not fill up to reach a maximum depth over 19 m, then the wetland areas may remain dry and disconnected from the reservoir surface water.

### 2.2 Sample Collection

#### 2.2.1 Fish collection

Fish were sampled from 11 locations within Cottage Grove Reservoir between June and August 2011, 2013, and 2014, and from seven downstream locations in the Willamette River in 2011. Fish were collected using a combination of beach seining and gill nets. Upon sampling, each fish was identified, measured to the nearest mm using a fish board, and frozen within 24 h in a uniquely labeled polyethylene zip close bag.

To estimate spatial and temporal variability in fish Hg concentrations, young-of-year (YOY) individuals (<100 mm) were sampled. The YOY fish collection focused on several species: Largemouth Bass (*Micropterus salmoides*), Northern Pikeminnow (*Ptychocheilus oregonensis*), Brown Bullhead (*Ameiurus nebulosus*), and Black Crappie (*Pomoxis nigromaculatus*). With the exceptions of Northern Pikeminnow, larger fish (>200 nm) of these species were also collected from the reservoir. Other adult fish species collected in the reservoir include: Yellow Perch (*Perca flavescens*), Bluegill (*Lepomis macrochirus*), and rainbow trout (*Oncorhynchus mykiss*).

#### 2.2.2 Water collection

Water samples were collected between January 2017 and October 2019 at five locations that receive mine-related Hg inputs and one location located upstream. At the time of sampling, Furnace Creek ran directly through mine tailings and represented a purely mine-influenced waterbody (remediation actions have occurred in this watershed after sampling was conducted). Samples from Furnace Creek were used as a mine-signature endmember during mixing-model analysis (described in the Supplementary Material). Water was collected from one location on Little River 1.5 km downstream of the inputs from Furnace Creek and then further downstream (9 km) at on the Coast Fork Willamette River (at the London USGS gaging station). Water was collected within the Cottage Grove Reservoir and directly downstream of the reservoirs in the Coast Fork Willamette River. In addition, one location was sampled upstream (~3 km) of the mine-site on Garoutte Creek to obtain a background Hg concentrations and endmember isotopic signatures.

For the total-Hg (THg) analysis, between 0.5 and 20 L of water were collected at each location depending on the THg concentration following standard clean-hands dirty hands techniques using a peristaltic pump and Teflon sample line. The larger sample volumes were collected for Hg stable isotope measurements. An inline filtration (0.45 μm disposable synthetic polymer filter capsule) was attached to the Teflon tubing for the collection of filtered samples (F-THg). All water samples were preserved immediately following collection (12 N trace metal grade hydrochloric acid (HCl); 0.5% acidification). Particulate-bound total-Hg (P-THg) was collected on the 0.45 μm filter and frozen following collection.

#### 2.2.3 Sediment and soil collection

Soil samples were collected in October 2016. The soils were collected from 11 locations at the mine site and from two nearby background locations (<1 km from the historic mine operations). At each sample location, 60 individual samples were collected (0–15 cm depth—after removing the surface leaf litter layer) using a disposable plastic scoop from three 100-m transects (one sample collected approximately every 5 m). These samples were combined into a single composite which was sieved to <1.5 mm. Samples were stored in new double Ziploc bags.

Sediment samples were collected in May 2019. Sediment core samples were collected from seven locations within Cottage Grove Reservoir and from one background location upstream of the reservoir on Williams Creek. The sediments were collected using a gravity corer (polycarbonate cores, 9 cm diameter) deployed from a boat in Cottage Grove Reservoir, while the background location was shallow enough where the core was directly inserted into the sediment by hand. Each sediment core was sectioned using an incremental core extruding apparatus such that the top 4 cm were removed using a plastic spatula and transferred to a 4oz glass sample container. All soils and sediments were kept on ice in the field and frozen until analysis.

### 2.3 Sample Analysis

#### 2.3.1 Mercury concentration analyses

At the USGS Forest and Rangeland Ecosystem Science Center in Corvallis, OR, each fish was rinsed with deionized water, blotted dry, measured for standard length (±1 mm), and weighed (±1 0.001 g). Fish were then dried to a constant weight (50°C for at least 48 h), cooled to room temperature in a desiccator cabinet, and weighed again (± 10.001 g). Dried samples (either whole fish or fillets) were homogenized using stainless steel scissors and a porcelain mortar and pestle and stored in a desiccator cabinet until mercury determination. Total Hg concentrations were measured for each fish sample following EPA method 7473 on a Milestone tri-cell DMA-80 Direct Mercury Analyzer (Milestone Inc., Shelton, Connecticut, United States). Quality assurance measures included analysis of two certified reference materials [either fish muscle tissue (DORM-4; National Research Council of Canada, Ottawa, Canada) or lobster hepatopancreas (TORT-3; National Research Council of Canada, Ottawa, Canada)], two system and method blanks, and two duplicates per batch of 40 samples. Recoveries averaged 104.3% ± 1.44% (*n* = 32) and 100.2% ± 1.7% (*n* = 52) for certified reference materials and calibration checks, respectively. Absolute relative percent difference for duplicates averaged 4.6% ± 1.0% (*n* = 39).

#### 2.3.2 Mercury stable isotopes

Mercury stable isotope measurements were performed by the USGS Mercury Research Laboratory (Madison, WI, United States) using the same methods as described in pervious publications (Lepak et al., 2019; Janssen et al., 2021a). A subset of all fish collected were analyzed for Hg stable isotopes, which are shown in Supplementary Table S1. Sediment and fish samples were acid digested (90°C for 8–10 h) prior to isotope analysis. The fish were acid digested using concentrated nitric acid (HNO_3_) followed by a bromine monochloride (BrCl) addition (10% v/v) and the sediments were digested using an aqua regia solution (3HCl:HNO_3_). Sample digests were diluted prior to isotope analysis (<10% acid and 0.5–1.2 ng mL−1 Hg). The certified reference materials (CRMs) used for sediment (IAEA SL-1 = 137 ± 3.1 standard deviation (1SD), 105% average recovery, *n* = 3 and NIST 1944 = 3,503 ± 62, 103% average recovery, *n* = 6), and fish (IAEA 407 = 213 ± 5, 1SD, 99% average recovery, *n* = 7) had acceptable concentration recoveries.

The Filtered-THg (F-THg) samples were processed using a modified resin pre-concentrated using an AG1-X4 (Chen et al., 2010; Štrok et al., 2014). Additional pre-treatment was needed to oxidize dissolved organic matter, which included an addition of 0.1% sodium persulfate followed by heating for 24 h at 55°C. After the initial sodium persulfate treatment, samples were oxidized with BrCl (1% v/v). The amount of THg within the sample was compared to the amount recovered post-processing to determine the percent recoveries for preconcentrated F-THg (107% ± 13%, 1SD, *n* = 6). Capsule filters containing suspended particulate matter were cut open using a heated nichrome wire and digested for particulate-THg (PTHg) in a 30% BrCl solution. The digests were heated for 14 days at 55°C before pre-concentration using a purge and trap method coupled to an oxidant trap (Janssen et al., 2019a). Percentage recoveries for P-THg were calculated by comparing the digestate to the final pre-concentrated oxidant trap (97% ± 7%, 1SD, *n* = 6).

A multicollector inductively coupled plasma mass spectrometer (MC-ICP-MS, Thermo Scientific Neptune Plus) was used for Hg stable isotope analysis. All solutions were diluted to 0.5–1 ng·mL^−1^ and <10% acid content. Samples were analyzed using standard-sample bracketing (Blum and Bergquist, 2007) with NIST 3133 and secondary standard NIST RM 8610 (UM Almaden) was analyzed every five samples, all solutions were matched within 10% Hg content and 1% acid content. Sample solutions were introduced into the instrument using stannous chloride reduction coupled to a custom-designed gas liquid separator, described elsewhere (Janssen et al., 2019a). For mass bias correction during analysis, a thallium standard (40 ng/ml, NIST 997) was simultaneously introduced into the gas liquid separator as an aerosol using an Apex-Q desolvating nebulizer. The MC-ICP-MS was tuned for optimal signal strength and stability for the analysis of Hg isotopes (1v^202^Hg~ 1 ng·mL^−1^ of Hg). Instrument accuracy and precision were assessed by the NIST 8610 values (δ^202^Hg = −0.52 ± 0.07, Δ^199^Hg = −0.02 ± 0.05, Δ^200^Hg = 0.01 ± 0.04, 2SD, *n* = 80), which agreed with the reference values. In addition, CRMs were analyzed within every sample batch (every five samples) to ensure digest efficiency. The secondary standard and the certified reference materials produced values in agreement with published literature (Blum and Bergquist, 2007; Estrade et al., 2010; Lepak et al., 2018) (see Supplementary Table S2).

#### 2.3.3 Carbon and nitrogen stable isotopes

Analysis of carbon (C) and nitrogen (N) stable isotope ratios was conducted at the Great Lakes Toxicology and Ecology Division laboratory in Duluth (MN). Fish were dried (50°C for >24 h) and homogenized prior to analysis. Analysis was conducted using a Costech 4010 Elemental Analyzer, coupled to a Thermo Delta Plus XP isotope ratio mass spectrometer. Analytical error was based on the standard deviation of replicate lab standards and was ±0.2‰ for δ^15^N and ±0.1‰ for δ^13^C. To correct for the negative bias in δ^13^C values associated with increasing lipid content, a mass-balance approach based on fish tissue C:N molar ratio was used to correct δ^13^C values for lipid content (expressed as δ^13^Clipid-free; fish C:N ratios ranged 3.6–5.2) (Hoffman et al., 2015).

#### 2.3.4 X-ray absorption fine structure (XAFS) analysis

The speciation of Hg in the soils and tailings samples was determined by extended X-ray absorption fine structure (EXAFS) using methods described in detail elsewhere (Eckley et al., 2021). The spectra from the samples were compared to reference materials that included cinnabar, metacinnabar, elemental Hg, HgCl_2_, Hg(NO_3_)_2_ (aq), Hg^2+^ -cysteine solution complex, Hg^2+^-histidine solution complex, and a Hg^2+^-citrate solution complex. The Hg L3-edge spectra were recorded at the Materials Research Collaborative Access Team (MRCAT) 10-ID line at the Advanced Photon Source (APS) operated by the U.S. Department of Energy (DOE) at the Argonne National Lab. The APS is a 7 GeV storage ring operating at 102 mA in top up mode. All samples and reference compounds analyzed (except elemental Hg) were initially ground to pass a 75 μm sieve. The materials were then mixed with polyvinylpyrrolidone and pressed into a 13 mm self-supporting pellet using a hand-held pellet press. The subsequent pellets were sealed between two pieces of Kapton^®^ tape and stored at 4°C prior to analysis. The elemental Hg sample was prepared placing a droplet of Hg^0^ between two polyethylene sheets and compressing the sheets together to disperse the droplet.

Incident X-rays were monochromatized using a cryogenically cooled Si (111) monochromator and a 250 mm long second crystal. A Si-coated flat harmonic rejection mirror was employed and a beam size of 400 μm × 400 μm was used. Incident beam energy was calibrated to the first derivative inflection point (12,284 eV) of elemental Hg. The monochromator was swept over the energy range 12,084 eV–13,084 eV at a rate of 1.5 eV per second. Data was collected in both fluorescence and transmission mode simultaneously. Fluorescence data was collected using a Lytle Box purged with Ar gas. A minimum of 10 and maximum of 20 scans were collected for each sample. Merging of the spectra was first performed using LARCH, followed by re-binning, background removal, and normalization using the Demeter XAS software package (Ravel and Newville, 2005; Newville, 2013).

#### 2.3.5 Statistical analyses for fish tissues

For the three primary fish species sampled (Largemouth Bass, Black Crappie, and Brown Bullhead) the fish were categorized as either YOY or adult based upon their lengths because the sampling design was heavily weighted towards the YOY size classes, and regression-based size-adjustments of Hg concentrations spanning the entire size spectra would introduce considerable bias. Prior to statistical analysis with the YOY fish, THg concentrations were size adjusted within each species using previously published methods (Eagles-Smith et al., 2016a; Eagles-Smith et al., 2016b). The larger size classes of fish were all very similar in size within species, so these samples did not necessitate size adjustment.

The THg concentrations (size adjusted for YOY individuals) were compared among species, size classes, and sites within Cottage Grove Reservoir using a general linear model (GLM) with the above factors as fixed effects. Because not all species were sampled across all years, the temporal variability was examined using a separate mixed-effects model that only included YOY Black Crappie and Largemouth Bass, which were sampled across three different years. This model included species, year, and a species × year interaction as fixed effects, and site ID as a random effect. The interaction term suggested that the two species may have had differing temporal responses (F_2,17.96_ = 2.95; *p* = 0.07); therefore, the separate models for each species were used. Finally, only YOY Largemouth Bass were sampled both within Cottage Grove Reservoir as well as downstream of the reservoir in the Willamette River. Size-adjusted concentrations between the reservoir fish and the downstream riverine fish were compared using analysis of variance. Calculations for the photochemical correction of δ^202^Hg using Δ^199^Hg (referred to as δ^202^Hg_COR_) are outlined in the Supplementary Material.

## 3 Results and discussion

### 3.1 Mercury levels in river and reservoir fish

Total Hg concentrations in fish from Cottage Grove Reservoir ranged from 0.02 to 1.13 mg/kg wet weight (ww) across all species, size classes, and sampling locations. While THg is being measured, it is assumed that most of the Hg that has accumulated in the fish is MeHg (Driscoll et al., 2007). Total Hg concentrations of fish larger than 200 mm (those commonly targeted for human consumption) averaged 0.55 ± 0.07 mg/kg ww, and 65% of individuals exceeded US EPA MeHg criterion for the protection of human health (0.30 mg/kg ww). Within the reservoir, THg concentrations differed among species/size class groupings (F_8,37.8_ = 31.5, *p* < 0.0001) and sampling sites (F_10,106.6_ = 71.0; *p* < 0.0001). After accounting for site differences THg concentrations were highest in adult Black Crappie and adult Largemouth Bass, and lowest in both adult (>200 mm) and YOY (<52 mm) Brown Bullhead (Supplementary Figure S1). There was substantial spatial variability in fish THg concentrations across the reservoir. The highest concentrations were measured in three locations close to the reservoir inflow and were > 4-fold higher than the lowest sites which were closest to the dam.

Whereas YOY Black Crappie showed no differences in THg concentrations among years (F_2,13.41_ = 1.45; *p* = 0.27), Largemouth Bass YOY THg concentrations differed between 2013 and 2014 (F_1,2.04_ = 22.61; *p* < 0.0001). Specifically, concentrations were 1.5x higher in 2013 (0.017 ± 0.01 mg/kg ww) than in 2014 (0.11 ± 0.01 mg/kg ww). With only a few years of data its difficult to determine the cause of the variations in fish THg concentrations between years; however, it may be related to differences in MeHg production impacted by reservoir water-levels and wetland connectivity, which vary between wetter and drier years (Willacker et al., 2016; Eckley et al., 2017).

**FIGURE 2:**
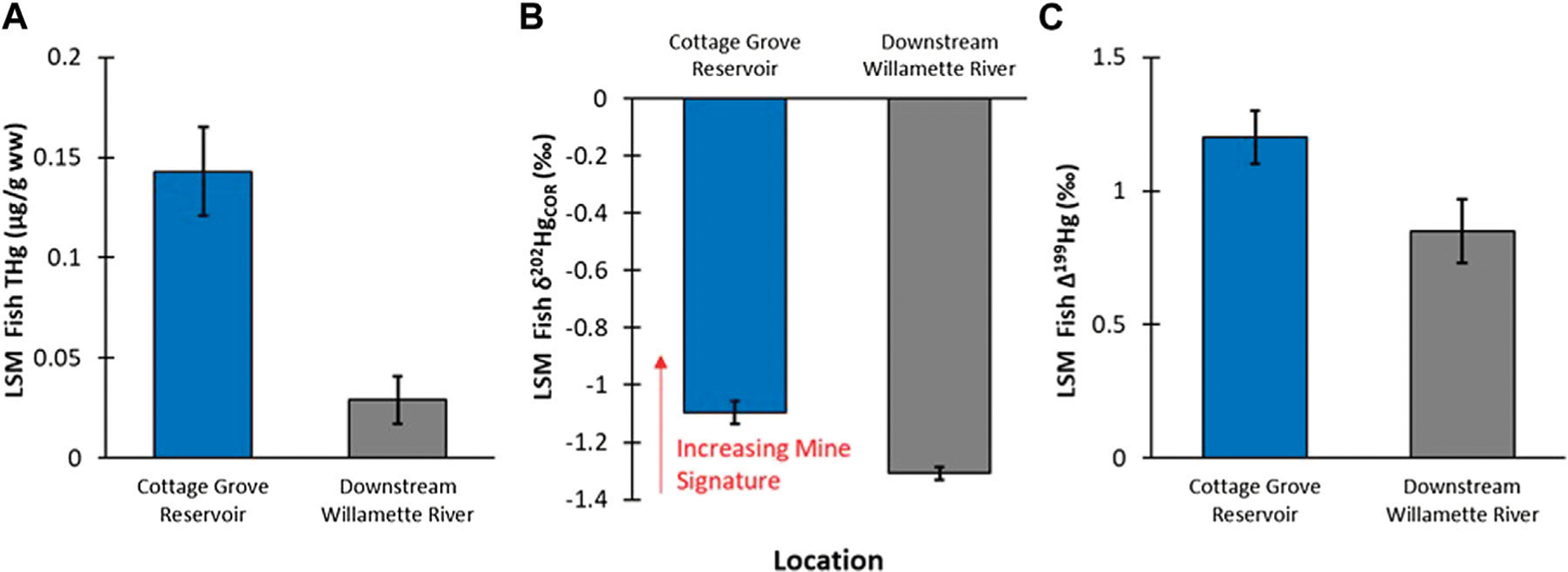
(**A**): Comparison of least square mean (LSM ± standard error—SE) of YOY Largemouth Bass fish tissue THg concentrations between Cottage Grove Resevoir (*n* = 14) and downstream sample locations in Willamette River (*n* = 38; fish length was a signficant covariate in the GLM analysis). (**B**): Comparison of LSM ±SE of YOY fish tissue δ^202^Hg compositions between Cottage Grove Resevoir and downstream sample locations in Willamette River (fish species was a significant covariate in the GLM analysis). (**C**): Comparison of LSM ±SE of YOY fish tissue Δ^199^Hg compositions between Cottage Grove Reservoir and downstream sample locations in Willamette River (fish species was a significant covariate in the GLM analysis).

Young-of-year Largemouth Bass was the only species collected from both Cottage Grove Reservoir and downstream (>90 km) of the reservoir in the Willamette River. Total Hg concentration in the reservoir fish were 4-fold higher than those from the Willamette River (F_1,9.41_ = 7.84; *p* = 0.02; Figure 2A). The higher Hg concentrations in the reservoir fish compared to downstream could be a function of higher Hg inputs from in the mine and/or enhanced methylation processes occuring in a lentic compared to lotic system (Eckley et al., 2015; Hsu-Kim et al., 2018).

### 3.2 The impact of sources and foodweb dynamics on fish mercury levels

**FIGURE 3:**
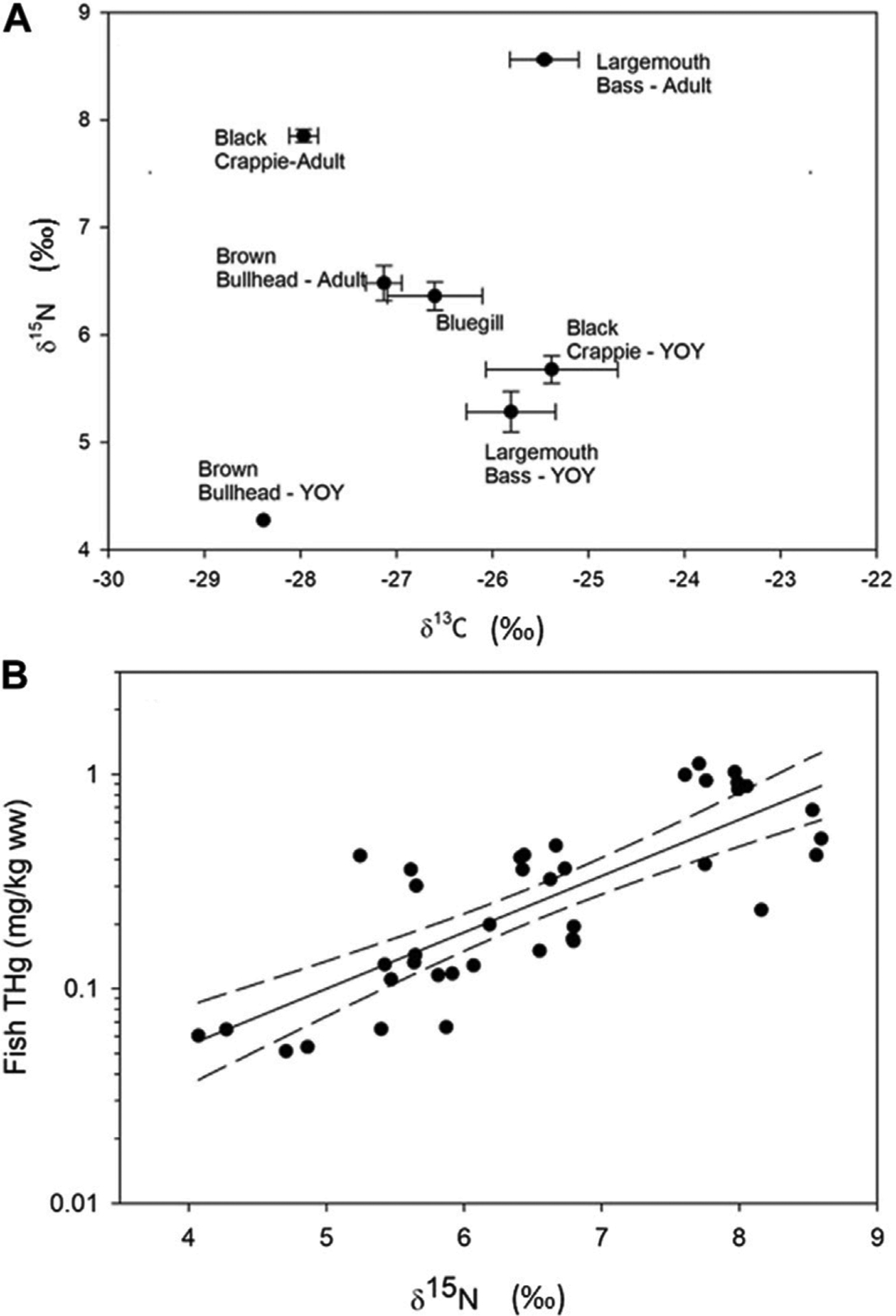
(**A**) shows the mean with standard error δ^15^N versus δ^13^C isotopic compositions in fish sampled in Cottage Grove Reservoir. (**B**) shows the relationship between tropic position (represented as δ^15^N) versus THg concentrations (*R*^2^ = 0.64*, p* < 0.0001; error bands represent 95% confidence intervals).

Mean δ^13^C_lipid-free_ values ranged from −28.4‰ in YOY Brown Bullhead to −25.4‰ in YOY Black Crappie (Figure 3A). This suggests a food web difference among species, shifting from increasing planktonic sources (low δ^13^C values) to increasing littoral sources (high δ^13^C values) (Hoffman et al., 2007). Young-of-year Brown Bullhead had the lowest mean δ^15^N value (4.27‰), whereas adult Largemouth Bass and Black Crappie had the highest mean δ^15^N values (8.56‰ and 7.85‰, respectively), indicating these fishes occupied the lowest and highest trophic positions, respectively (Figure 3A). Total Hg concentrations in fish fillet tissues were highly correlated with δ^15^N values (F_1,37_ = 65.3; *p* < 0.0001; *R*^2^ = 0.64; Figure 3B), increasing 0.556 mg/kg ww from a δ^15^N value of 4‰–8‰.

The higher YOY Largemouth Bass Hg concentrations in the reservoir compared to the free-flowing section of the river (Figure 2A) may be related to the higher Hg concentrations in water and sediment within the reservoir compared to downstream (Hope, 2006; Eckley et al., 2015); but may also be related to changes in Hg methylation and biomagnification related to shifting from a reservoir environment to a free-flowing river (Willacker et al., 2016; Hsu-Kim et al., 2018). However, because the comparison is being made on YOY fish of the same species, the impact of differences in foraging behavior would be very limited.

### 3.3 Characterizing the mercury isotopic composition in an upstream source area

**FIGURE 4:**
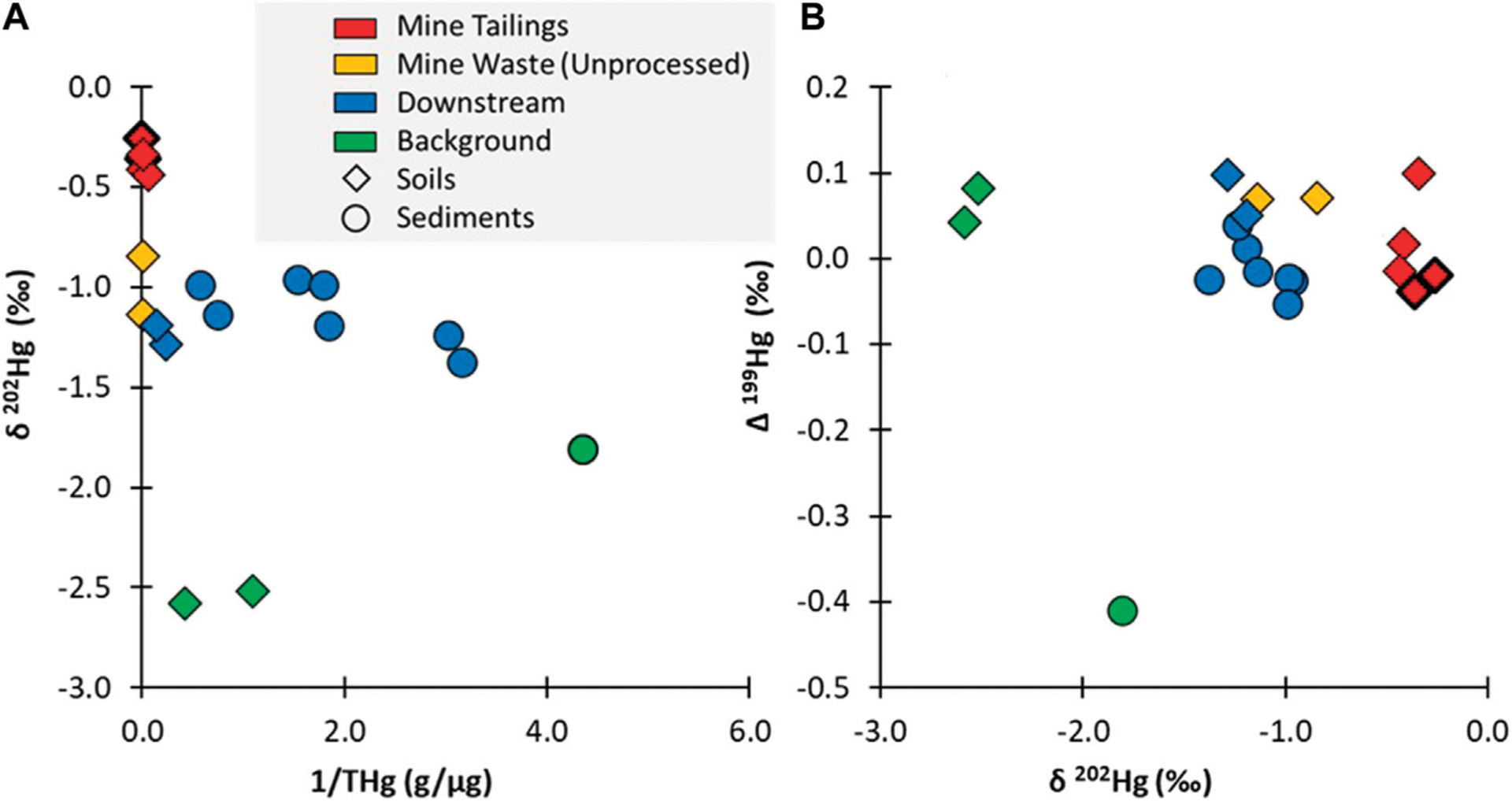
(**A**) showing the δ^202^Hg values plotted against the inverse THg concentration (μg/g); and (**B**) showing the Δ^199^Hg versus δ^202^Hg values. Note: all soil samples are plotted as diamonds and all sediment samples are plotted as circles. The tailings samples from the old furnace are distinguished from the newer furnace with a heavier black outline.

Background soil samples collected from outside of the mining area had high mean Hg concentrations (1.6 μg/g) reflecting that the mine is situated within a naturally geologically enriched area (Figure 4A). The δ ^202^Hg values from the background soils were quite low (mean: −2.55‰), indicating that they were not influenced by direct inputs from a mining/industrial source as seen in other contaminated sites (Eckley et al., 2020). However, it is noted that highly negative values for δ^202^Hg have been observed in cinnabar ore as well as other Hg containing ore deposits (Smith et al., 2005; Smith et al., 2008). Given the geology of the area and the high Hg concentrations within the background soil, it is likely that the geogenic background of the region is highly depleted with regard to the δ^202^Hg isotopic signature. The background sediment sample was collected from outside of the geologically enriched zone and has a much lower THg concentration (0.23 μg/g) and a lower δ^202^Hg value (−1.81‰). The difference in isotopic compositions between these two types of background media may be related to differences in watershed processing of the geogenic source that impact the δ^202^Hg isotopic signature (e.g., redox shifts during mineralization, elemental versus oxidized Hg inputs from air versus precipitation, etc.). It is difficult to ascertain potential processing or source differences using δ^202^Hg because of the large overlap between values for ore material (Smith et al., 2005; Smith et al., 2008)and background reference values derived predominantly from atmospheric deposition (Eckley et al., 2020). However, examining Δ^199^Hg values indicates there is more potential influence from terrestrial processing within the sediments due to lower values (soils: 0.06‰; sediment: −0.41‰) (Figure 4B). It is noted that no substantial differences were observed in Δ^200^Hg values between the samples. Typically, negative Δ^199^Hg values have been attributed to foliar uptake of gaseous elemental Hg (Demers et al., 2013; Enrico et al., 2016), which may indicate the sediments are reflective of mixed Hg sources (geogenic and atmospheric deposition), supported by the lower concentrations, or a subset of geogenic Hg pools are integrated and processed within the terrestrial environment, such as through soil re-emission.

Compared to the background samples, the mine tailings had a distinct δ^202^Hg value (−0.35‰ ± 0.05‰), which is over a span of two ‰ different from the background soils and is more aligned with direct point sources measured at other contaminated sites (Eckley et al., 2020). The Hg isotopic composition of the tailings that originated from the newer versus the older furnaces had similar δ^202^Hg values (old furnace tailings: −0.31‰; new furnace tailings: −0.40‰), indicating that there were no distinguishable differences between these two sources. There was a larger difference in the δ^202^Hg value (> 0.5‰) when comparing the unprocessed mine waste (−0.99‰), indicating that the process of roasting the ore resulted in fractionation and contributed to the distinct isotopic composition of the tailings, as seen in other studies (Smith et al., 2014). The EXAFS data of the newer and older tailings and unprocessed mine waste confirms that there are distinctions in the Hg speciation in these materials (Supplementary Table S3). The tailings from the newer furnace is entirely composed of cinnabar; whereas the less efficient extraction process in the older furnace contained some metacinnabar. The unprocessed mine material is unique in that it contains Hg bound to organic material, which would have been easily removed during the ore-processing in a furnace.

**FIGURE 5:**
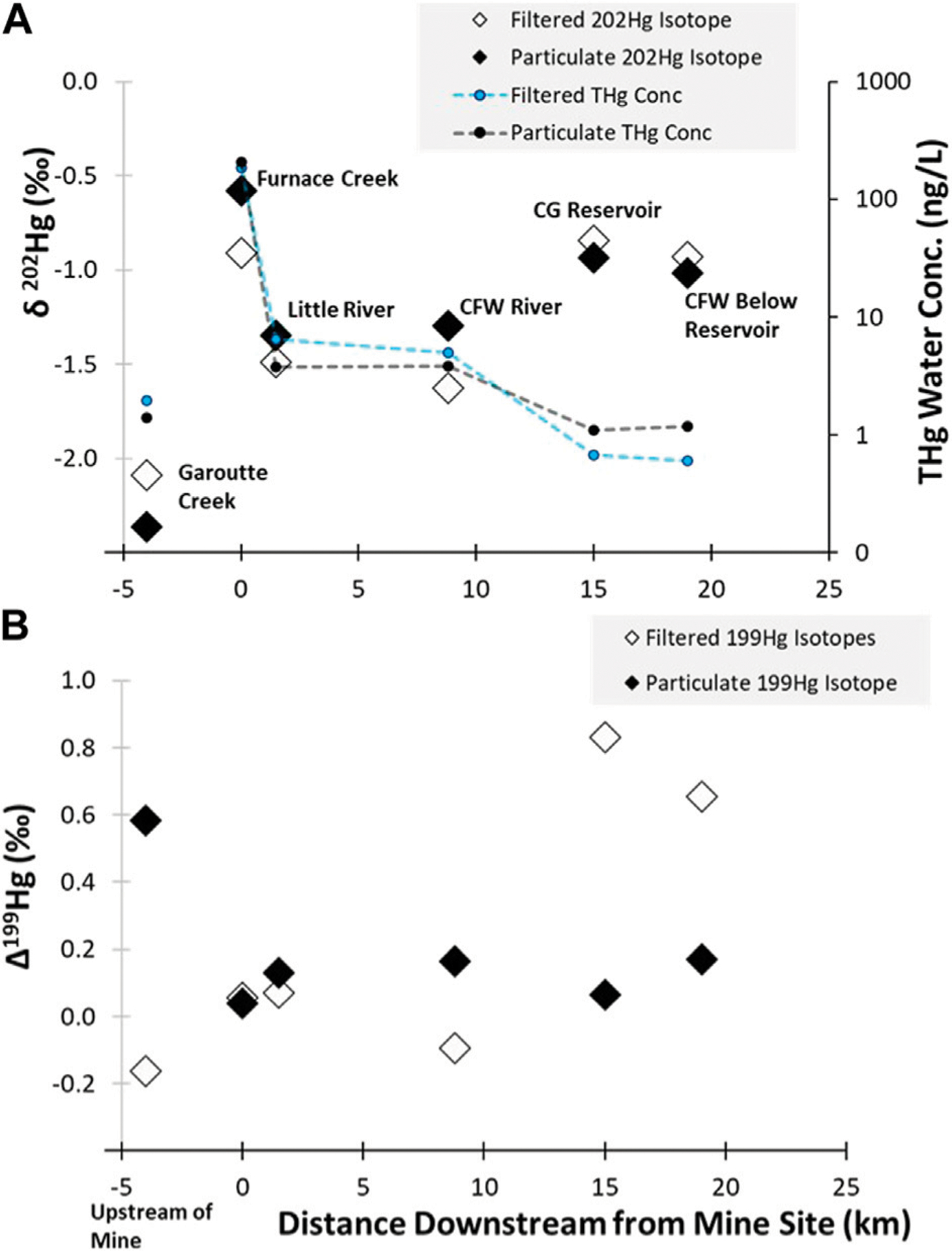
(**A**) shows the THg concentrations and δ^202^Hg values in water samples for particulate and filtered phases and (**B**) shows this relationship for the Δ^199^Hg values in water. The background samples were collected approximately 3 km upstream of the mine in Garoutte Creek (−3 km on the graph). The 0 km location marks the location of the Black Butte Mine and Furnace Creek. Furnace Creek flows into Little River, which was sampled 1 km downstream of the mine. Little River flows into the Coast Fork Willamette (CFW) river and was sampled 9 km downstream of the mine. The Cottage Grove (CG) reservoir is 15 km downstream of the mine and at 19 km downstream a sample was collected below the reservoir on the CFW River. Note that the secondary *y*-axis in (**A**) is plotted on a log-scale. Uncertainty of measurements was assessed by the 2SD of processing standard NIST 3133, which was 0.09‰ for both δ^202^Hg and Δ^199^Hg.

The δ^202^Hg value from water draining the mine site in Furnace Creek showed a similar isotopic signature as the tailings samples (particulate δ^202^Hg: −0.58‰; filtered δ^202^Hg: −0.89‰), which were much more enriched than the background stream samples (particulate −2.36‰; filtered: −2.09‰; Figures 4, 5A). The δ^202^Hg value in these water samples shows a ~0.30‰ spread between the dissolved and particulate fractions, though the directionality differs between the two sites. Previous work has proposed that the difference between the filtered and particulate phases is a function of Hg sources, with dissolved phases represented atmospheric deposition while particulates are more similar to terrestrial runoff (Campeau et al., 2022). While this is possible, there was no evidence of differences in the Δ^200^Hg ratios between the phases, which should be able to easily characterize precipitations pools of Hg (Kwon et al., 2020). Furthermore, the Δ^199^Hg from the background water samples had a larger span in values between the filtered (−0.16‰) and particulate phases (0.58‰), which may indicate different levels of photochemical processing of the same source. It is unclear why there is higher photochemical processing (as indicated by Δ^199^Hg) on the particulate matter rather than in the dissolved phase, but it indicates that environmental processing could have occurred during mobilization and sorption reactions resulting in the disconnect between the phases (Figure 5B). Further examination would be needed to assess if different Hg sources (e.g., precipitation and dry deposition) or *in situ* processing is contributing to the dissolved and particulate Hg isotope ratios observed.

### 3.4 Tracking the mercury isotopic composition in downstream water and sediment

Downstream sediment samples collected from the Cottage Grove Reservoir had higher δ^202^Hg values compared to background samples (Figure 4A). While all the reservoir sediment samples were enriched in ^202^Hg, there was some variability in this composition, which varied from −0.96‰ to −1.38‰, reflecting variations in sediment depositional patterns in the reservoir (see Supplementary Figure S2). For example, the two locations with the lowest δ^202^Hg values were both located in wetlands along the edge of the reservoir, which are less impacted by the deposition of suspended particulates from the inflowing water. Enrichment in ^202^Hg corresponded with increasing sediment THg concentration, indicating that particles mobilized from the mine site are a likely contributor to elevated THg in the reservoir (Supplementary Figure S3).

Mercury concentrations decrease with distance moving downstream from Black Butte Mine (Figure 5). While Furnace Creek, which drains through the mine site, contains elevated Hg concentrations (whole water THg: 391 ng/L), the creek had a very low discharge (0.0028 m^3^/s) and the Hg concentrations are quickly diluted (whole water THg: 10.3 ng/L) when it empties into Little River which has a much larger discharge (2.5 m^3^/s). This dilution of the mine -related Hg inputs with low Hg content water from background sources results in a shift in the δ^202^Hg values of both the filtered (−1.49‰) and particulate bound fractions (−1.35‰). However, mixing model calculations (see details in the Supplementary Material) indicate that even after being diluted by the larger downstream flows, 51% of F-THg and 63% of P-THg originated from mine-related sources. In addition, the Δ^199^Hg value for the P-THg downstream of the mine site is consistent (0.13‰ ± 0.2‰) and similar to what was measured directly draining the tailings areas (0.04‰), confirming that the mine is an important source of Hg water further downstream of the mine. The discharge of the river is further increased 9 km downstream of the mine site after Little River combines with Big River to form the Coast Fork Willamette River (9.3 m^3^/s); however, the Hg concentrations (whole water THg: 8.9 ng/L) is only slightly lower than what was measured in Little River. The δ^202^Hg values in dissolved and particulate samples are similar to the upstream site at Little River (3 km downstream from the site), indicating there is still a significant mining influence present. Dissolved and particulate phases do show divergence from one another in δ^202^Hg and Δ^199^Hg values, indicating that there may be integration of other runoff sources, potentially indicated by the negative Δ^199^Hg in the dissolved phase, or *in situ* processing, such as photochemistry. The more stable Δ^199^Hg values also indicates that the particles are more recalcitrant to environmental processing compared to Hg in the dissolved phase. Values in both matrices are still well above the established geogenic background soils in the region and approximately 40% of dissolved Hg and 70% of particulate Hg could be attributed to mine-related releases. (Figure 5A).

The water samples from the background/upstream location (Garoutte Creek) shows a larger Δ^199^Hg value associated with the suspended particulate samples compared to the dissolved phase (Figure 5B). This may result from atmospheric Hg sources that efficiently sorb to the particles or that the particles represent a pool with a longer exposure time in comparison to the filtered water samples which is more transient. However, further investigation would be needed to provide a better understanding of the factors contributing to the upstream isotopic signature in water.

Once the water reaches the reservoir, there is an interesting divergence in the trends in Hg water concentrations and isotopic compositions. The whole water THg concentrations in and below the reservoir continue to decrease to levels that are similar to the background/upstream location (all < 4 ng/L). However, the Hg isotope ratios showed an increase in the δ^202^Hg value in both the filtered (reservoir: −0.84‰; below reservoir: −0.93‰) and particulate phases (reservoir: −0.94‰; below reservoir: −1.02‰), which suggests that the Hg had had a greater contribution from the mine than the lotic locations closer to the mine. It is worth noting that the dissolved phase also had higher Δ^199^Hg value (0.83‰ in the reservoir and 0.66‰ downstream of the reservoir), likely due to enhanced photochemical degradation of inorganic Hg in the reservoir, which likely explains the elevated δ^202^Hg values. However, the particulates do not show any elevated Δ^199^Hg values, indicating that there is no change in photochemical processing between the riverine particles and those collected in the reservoir. This observation indicates that the particulates are representative of a distinct source in the reservoir, different than what was observed in the lotic locations, whereas the dissolved Hg is related to riverine sources that are undergoing transformations in the reservoir. A potential source of mine-related Hg in and downstream of the reservoir is the reservoir sediment. The river system directly downstream of the mine is relatively high-energy and particles mobilized from the mine mostly stay in suspension. These particles are deposited/accumulated in the sediment of the reservoir (Curtis et al., 2013), which may also act as an ongoing secondary source to the reservoir water-column—especially when the reservoir is in low-pool conditions and the river cuts through previously deposited sediments.

### 3.5 Hg stable isotopes in fish

It is challenging to compare Hg isotopic composition in fish tissue to abiotic matrices such as water and sediments due to the net process of methylation as well as photodemethylation. To assess potential differences between the reservoir and the river, we opted to directly compare fish tissues from the same species and age classes (YOY). While this allowed for an apt biological comparison, there were still obvious differences between the reservoir and riverine fish related to the extent of photochemistry (see plots of δ^202^Hg versus Δ^199^Hg values in Supplementary Figures S4, S5). All fish displayed positive Δ^199^Hg values, which is typical of photodegradation of MeHg (Δ^201^Hg/Δ^199^Hg slope of all fish = 1.31, *r*^2^ = 0.996, *n* = 75) (Bergquist and Blum, 2007). The Δ^199^Hg values in fish from Cottage Grove Reservoir were significantly higher (1.2‰) than those from downstream (0.85‰, *p* = 0.018; Figure 2C). Higher Δ^199^Hg values in reservoir fish is reflective of photochemical processes, which can often be more pronounced in lentic relative to lotic systems due to higher water clarity and euphotic feeding (Lepak et al., 2018). Due to the fact that photochemical processes also produces MDF, δ^202^Hg values are changed (Bergquist and Blum, 2007), so photochemical corrections (denoted as δ^202^Hg_COR_) need to be applied to meaningfully compare fish Hg stable isotope ratios (Gehrke et al., 2011; Janssen et al., 2019b; Rosera et al., 2022). Whole body YOY fish from Cottage Grove Reservoir had a significantly higher δ^202^Hg_COR_ value compared to fish collected further downstream (Figure 2B); which supports the interpretation that the mine-influence on Hg concentrations in YOY fish is larger in the reservoir and decreases downstream in the Willamette River. There are caveats to this approach; specifically, there could be different methylation environments (Rosera et al., 2022) within the lotic and lentic habitats or that photochemical corrections may vary due to differences in organic matter (Chandan et al., 2015). Nevertheless, this data provides the first evidence that there is a difference between the Hg sources bioaccumulated in the reservoir and lower Willamette River.

**FIGURE 6:**
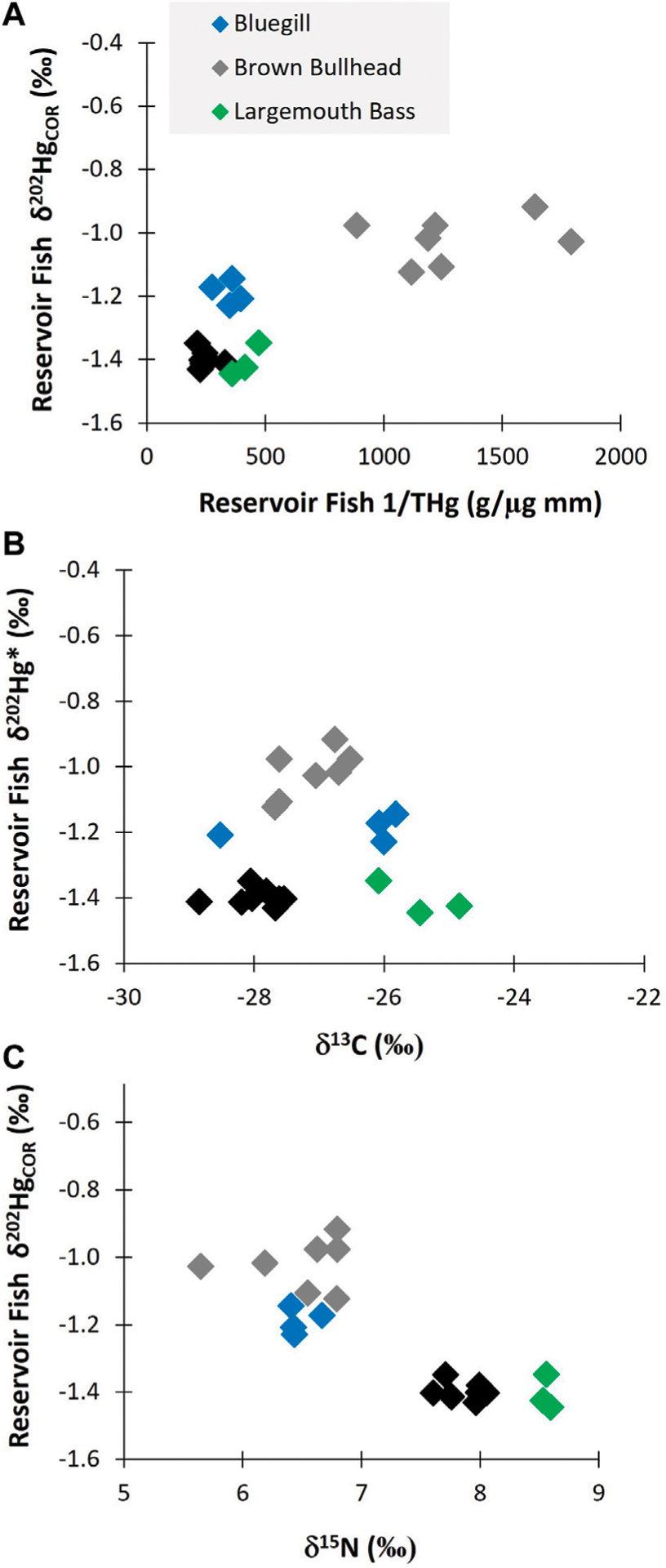
(A) shows the δ202HgCOR values for different adult fish species as a function of length normalized THg concentrations in Cottage Grove Reservoir. (B) shows the δ202HgCOR values for different fish species as a function of the δ 15N value. (C) shows the δ202HgCOR values for different fish species as a function of the δ13C value.

Within the reservoir, the degree of Black Butte mine-related Hg (as represented by δ^202^Hg_COR_) varied with tissue THg concentration (Figure 6A). These results show that the fish with the highest THg concentrations were less influenced by mine sources than fish with lower THg concentrations. The relationship between fish THg concentrations and δ^202^Hg_COR_ value is significantly (*p* < 0.01) influenced by differences in fish species, which was the primary driver of differences in isotopic composition (Supplementary Figures S5, S6). Among the fish species, Brown Bullhead had significantly lower Δ^199^Hg values (0.77‰ ± 0.12‰) than Largemouth Bass, Bluegill, and Black Crappie (mean of all three species: 1.57‰ ± 0.15‰). The lower Δ^199^Hg value in Brown Bullhead indicates that the source of Hg in their diet has more a benthic/sediment source than the other fish species, which appear to be more influenced by Hg uptake from the water-column (Supplementary Figure S7). This is consistent with the δ^13^C data, such as the Brown Bullhead having δ ^13^C values that are intermediate between Black Crappie and Largemouth Bass (Figures 3A, 6B). Brown Bullhead δ^13^C values (−25 to −28‰) are typically associated with riverine food web pathways based on terrestrial-derived, sediment sources, which is distinct from littoral-based food web (e.g., δ ^13^C > −22) and a phytoplankton based food web (e.g., δ^13^C > −30) (Hoffman et al., 2007). As such, Bluegill is expected to be the most similar in food web pathways to Brown Bullhead, Black Crappie is interpreted to have a greater contribution from phytoplankton, and Largemouth Bass to have a greater contribution from littoral sources. Thus, differences in δ^13^C values between fish species in the reservoir support the interpretation that food web pathways, Δ^199^Hg values, and δ^202^Hg_COR_ values are related, such that there is higher influence of mine-sourced Hg in fish feeding in a more sediment-based food web and less so in planktonic and littoral-based food webs.

The δ^202^Hg_COR_ values had a negative relationship with δ ^15^N values (Figure 6C). This indicates that mine-related Hg burdens vary due to differences in trophic position, habitat usage and Hg cycling differences within habitats. The Hg isotopes shows that bullhead are disproportionally impacted by mine-related Hg, which is likely due to their comparatively greater preference for benthic habitats. The shift in diet/foraging behavior that is causing the decrease in mine-related influence on the higher trophic position fish is uncertain. Notably, the two species with the lowest δ^15^N values also had intermediate δ^13^C values, indicating the potential for both trophic position and food web pathway to contribute to bioaccumulation of mine-sourced Hg.

Overall, the Hg isotopic composition data indicate that fish in Cottage Grove Resevoir are impacted from Hg releases associated with Black Butte mine (Figures 2, 6). However, the amount of mine-related Hg does not correlate with the total concentration of Hg in the fish and is more related to fish species and their different foraging behaviors (Figures 3, 6; Supplementary Figure S6). These results show that the lower trophic position species (i.e., Brown Bullhead) with a slightly larger dietary connection to the sediment could be more impacted by mine-related Hg sources; even though they have lower tissue THg concentrations than other fish species (i.e., Black Crappie). Whereas the sediment and surface water data for the reservoir indicate both media contain a large proportion of mine-related THg (see Figures 4, 5), the mine-related inorganic Hg may be much less available for methylation than Hg from other sources. This is consistent with findings from other contaminated sites, where the % MeHg decreases as the THg concentration increases (Eckley et al., 2020) and suggests the source of MeHg that the fish are exposed to differs from the primary source of THg in water and sediment.

We conclude that the examination of Hg transport from the Black Butte mining site has indicated that mine-derived Hg was transported downstream to lotic and lentic habitats (Curtis et al., 2013; Eckley et al., 2015). Mercury stable isotopes revealed that mining sources could be connected in different environmental compartments, but also showed there are a myriad of processes, specifically in waters and fish tissue, that can also be occurring. This work served as a first step to elucidating fate and transport of mine-related Hg to Cottage Grove Reservoir and the Willamette River.

## Supplementary Material

Supplementary Material

## Data Availability

The raw data supporting the conclusion of this article will be made available by the authors, without undue reservation.
