# Supplementary material for: Using mercury stable isotope fractionation to identify the contribution of historical mercury mining sources present in downstream water, sediment and fish
Source: Front Environ Chem. Author manuscript; Available in PMC 2023 Jun 15. (PMC10269370; doi:10.3389/fenvc.2023.1096199)
Supplement: Supplementary Material [file NIHMS1903652-supplement-Supplementary_Material.docx]

**Supporting Information for “Using mercury stable isotope fractionation to identify the contribution of historical mercury mining sources present in downstream water, sediment and fish.”**

**Authors:** Chris S. Eckley, Collin Eagles-Smith, Todd P. Luxton, Joel Hoffman, Sarah Janssen

**Affiliations:**

1. U.S. EPA Region 10, Seattle, WA USA
2. U.S. Geological Survey, Forest and Rangeland Ecosystem Science Center, Corvallis, OR, USA
3. US EPA ORD, Center for Environmental Solutions and Emergency Response, Cincinnati, OH, USA
4. U.S. EPA Office of Research and Development, Center for Computational Toxicology and Exposure, Great Lakes Toxicology and Ecology Division, Duluth, MN, USA
5. U.S. Geological Survey, Mercury Research Lab, Upper Midwest Water Science Center, Madison, WI, USA

**Supplementary Methods**

**Photochemical Corrections**

Photochemical corrections of δ^202^Hg were performed as set forth in previous publication(Blum and Bergquist, 2007; Gehrke et al., 2011; Sherman and Blum, 2013) using a slope determined from bench top experiments for photochemical demethylation (Bergquist and Blum, 2007). A slope of 2.43 was utilized for consistency since the average dissolved organic carbon (DOC) across sites was well below 5 mg/L(Eckley et al., 2015) . Corrections were performed as:

δ^202^Hg_COR_ = δ^202^Hg_fish_ – (Δ^199^Hg_fish_/2.43) (Equation 1)

Previous assessments have shown that using the photochemical slope for higher dissolved organic carbon conditions (4.79) did not result in substantially different corrected values (Janssen et al., 2019), hence fish from the reservoir and river were treated similarly.

**Binary Mixing Model Calculations**

The relative contributions of Hg from Black Butte Mine associated with suspended sediment was estimated using a binary mixing model. It is assumed that the Hg isotopic signature from two independent end-members mixes conservatively.

The Hg isotope balance is calculated as:

δ^202^Hg_mix_ = f_1_ δ^202^Hg_1_ + f_2_ δ^202^Hg^2^  (Equation 2)

δ^202^Hg_1_, δ^202^Hg_2_, and δ^202^Hg_mix_ represent the Hg stable isotopic compositions of end-members 1 and 2 and their mixture, respectively, and f_1_ and f_2_ represent the proportions of end-members 1 and 2 in any mixture. Assuming that f_1_ + f_2_=1, solving for f_1_ gives:

f_1_ = (δ^202^Hg_mix_ − δ^202^Hg_2_)/( δ^202^Hg_1_ – δ^202^Hg_2_) (Equation 3)

Equations 1 and 2 were applied to water samples collected downstream of the Black Butte Mine. The mine-related end-member (i.e. δ^202^Hg_1_) was based on signature from Furnace Creek (suspended sediment: -0.58 ‰; dissolved: : -0.91 ‰) The background end-member (i.e. δ^202^Hg_2_) was based on water from Garoutte Creek located upstream of the mining-area (suspended sediment: -2.36 ‰; dissolved: : -2.09 ‰).

**Supplementary Figures and Tables**


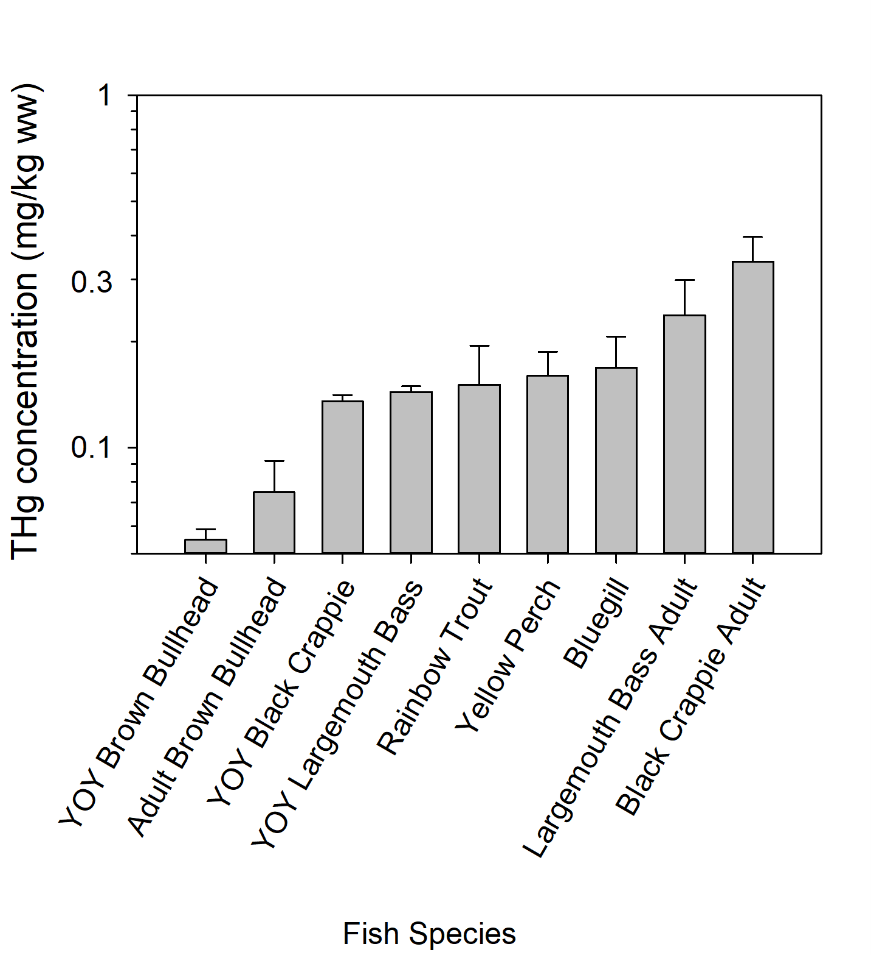


**Figure S1**. Mean with standard error total mercury (THg) concentrations in fish from Cottage Grove Reservoir, OR, USA. Details on sample sizes of each fish species provided in Table S1 in the SI.


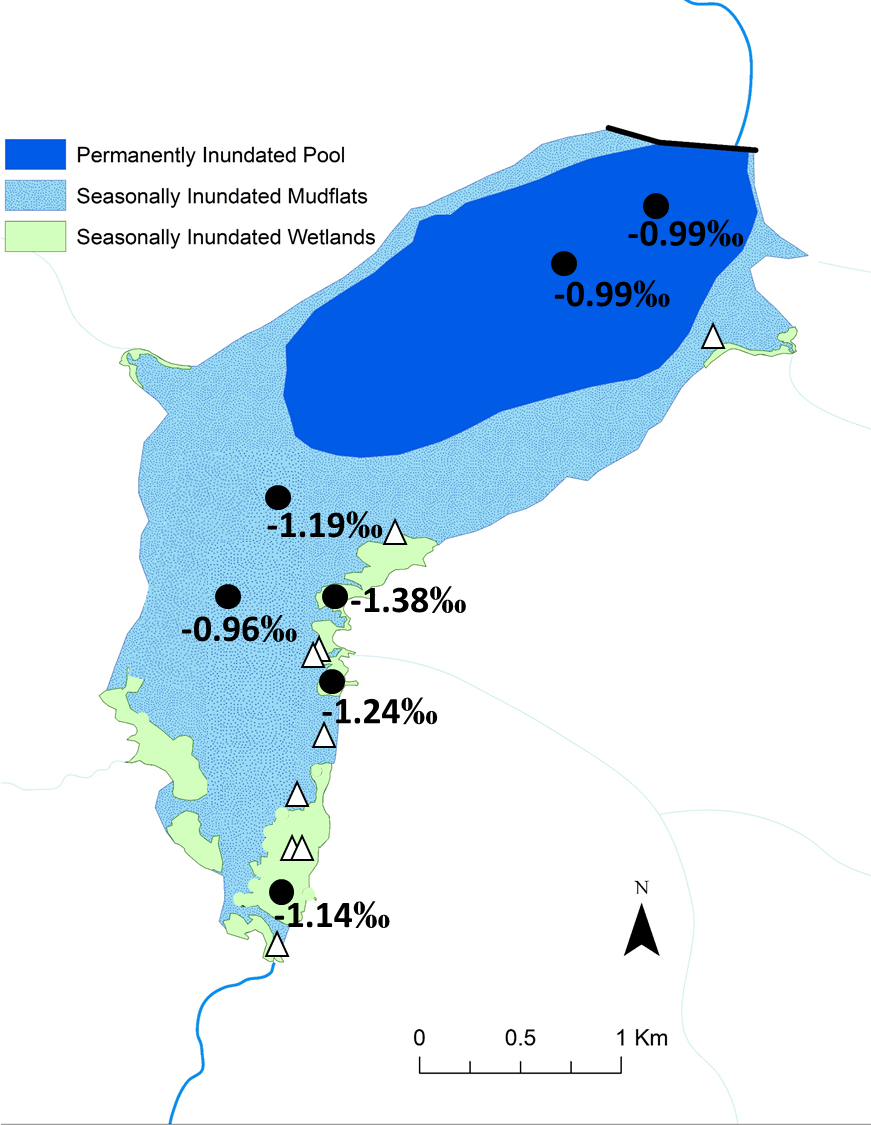


**Figure S2**. Map of Cottage Grove Reservoir. The black circles show the location of the sediment sample collections and the associated δ^202^Hg signature. The white triangles show the locations where fish samples were collected.


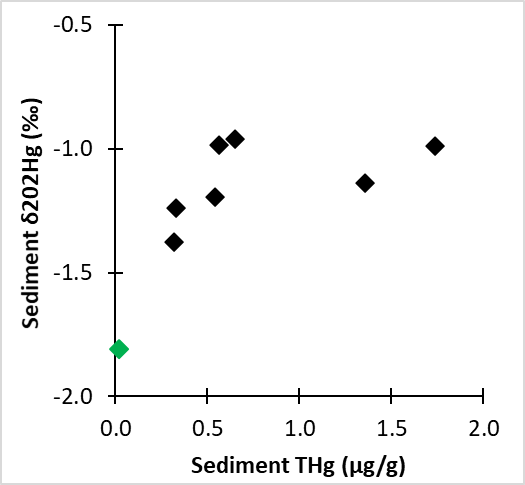


**Figure S3**. Graph of the sediment total mercury (THg) concentration versus δ^202^Hg signature in Cottage Grove Resevoir and background location (shown with green symbol).


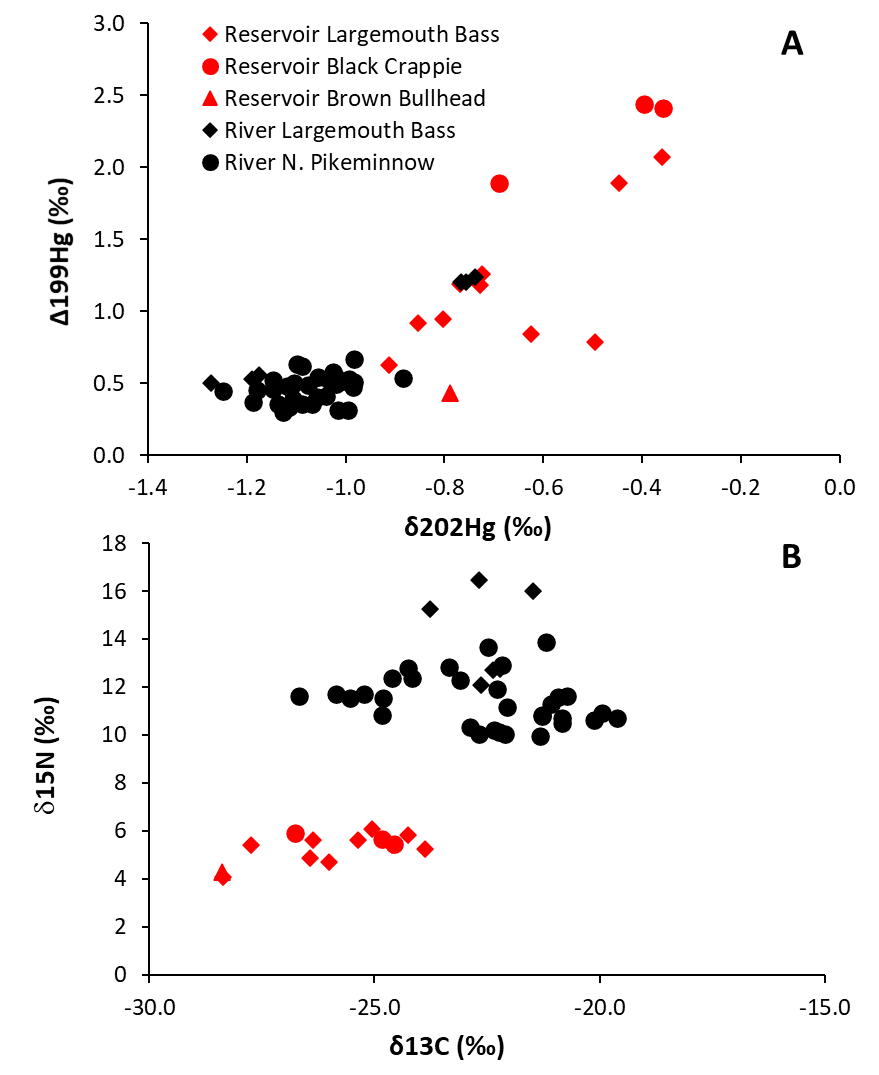


**Figure S4.** Graph A: Hg isotope biplot of whole-body young-of-year (YOY) fish collected from Cottage Grove Reservoir (red symbols) and downstream of the reservoir in the Willamette River (black symbols). Higher photochemistry is observed in the fish collected from Cottage Grove. This trend likely reflects waterbody type with more open water regions exhibiting more photochemical demethylation in biological tissue. Graph B: Carbon and Nitrogen isotope biplots of whole-body young-of-year (YOY) fish collected from Cottage Grove Reservoir (red symbols) and downstream of the reservoir in the Willamette River (black symbols).


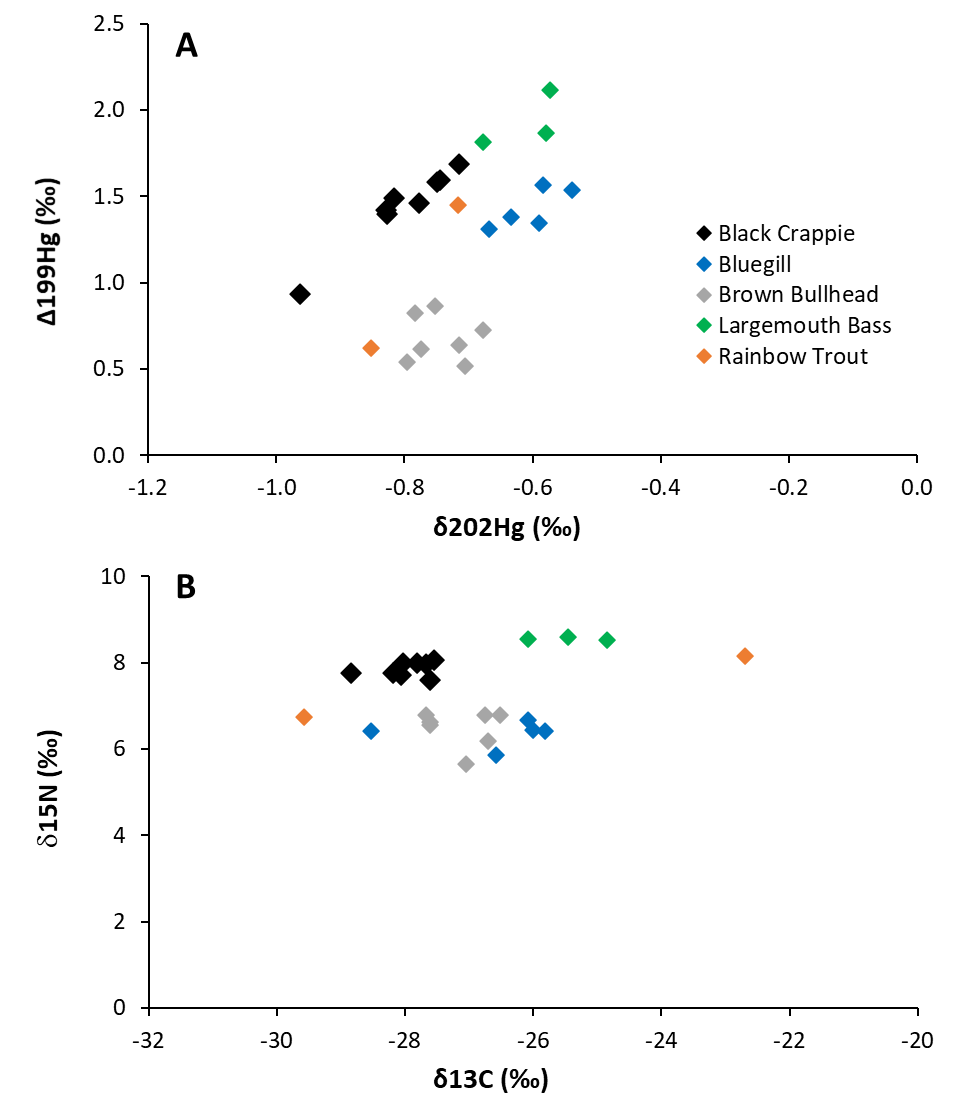


**Figure S5.** Graph A: Hg isotope biplot of fish fillets collected from Cottage Grove Reservoir, with different fish species plotted in a different color. Graph B: C and N isotope biplot of fish fillets collected from Cottage Grove Reservoir, with different fish species plotted in a different color.

**Figure S6**. Least square mean (LSM) fish tissue δ^202^Hg_COR_ ratios with standard error for each fish species collected in Cottage Grove Reservoir. The LSM values were obtained from generalized linear model (GLM) analysis with fish tissue total mercury (THg) concentration normalized to fish length as a non-significant covariate (p=0.18, degrees of freedom (df)=18); however, differences in fish species were significant (p<0.001, df=18). The fish species cluster into three different groups with significantly different concentrations: group 1: Largemouth Bass and Black Crappie; group 2: Bluegill; group 3: Brown Bullhead.


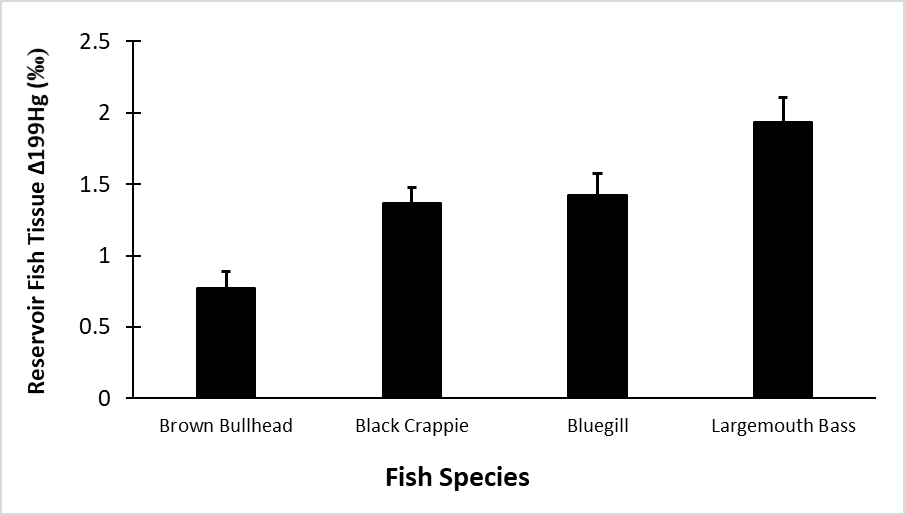


**Figure S7**. Figure showing the least square mean (LSM) ± standard error of the Δ^199^Hg signature for reservoir fish tissue samples. The LSM values are based on generalized linear model analysis with fish length as a covariate (p=0.13, df=18). There were significant differences in the Δ^199^Hg signature (p<0.001) between fish species—specifically, the Brown Bullhead was significantly lower than the Black Crappie, Bluegill, and Largemouth Bass; with all other fish species not showing significant differences.

**Table S1.** Fish samples selected for Hg isotope analysis with location, sample type, species and standard length.


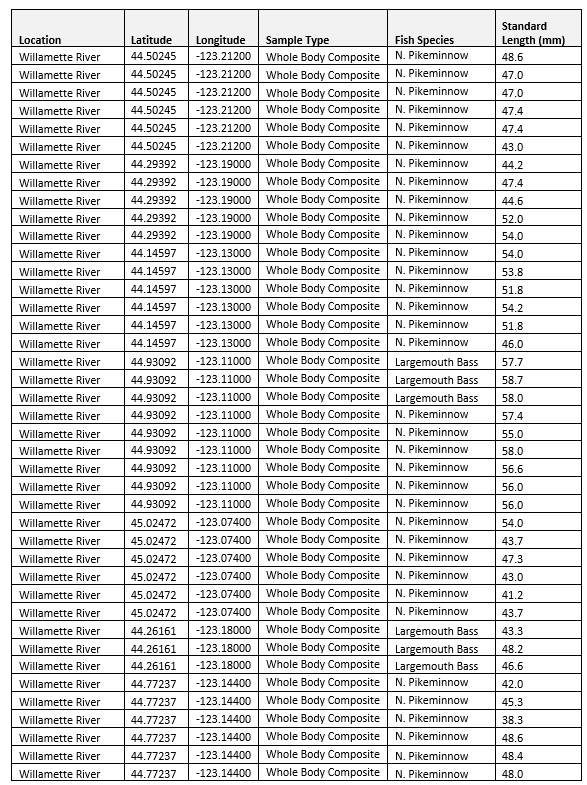


**Table 1 continued:**

**
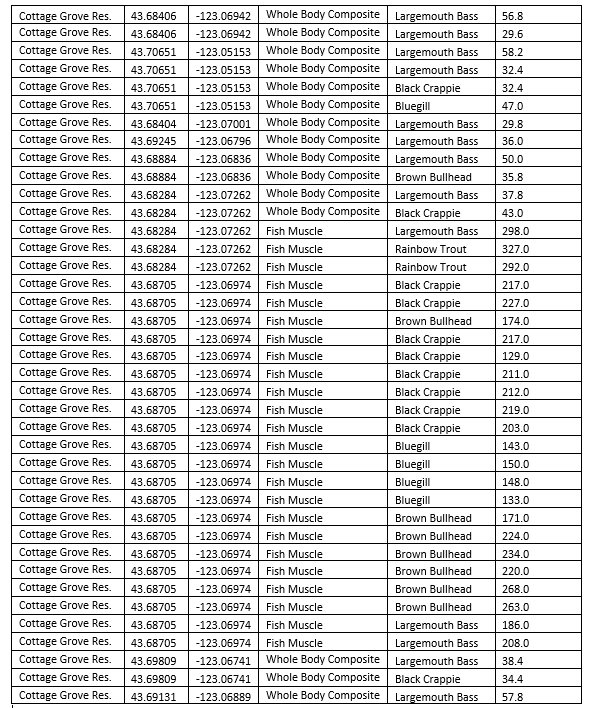
**

**Table S2**: Mercury stable isotope quality control and assurance for certified reference materials. Certified reference materials from the International Atomic Energy Agency (IAEA) and the National Institute of Standards and Technology (NIST) were analyzed alongside samples to ensure accuracy and precision. No certified reference materials were available for large volume water samples so NIST 3133 dilutions processed alongside preconcentration methods, percentage recovery refers to post-processing recoveries.


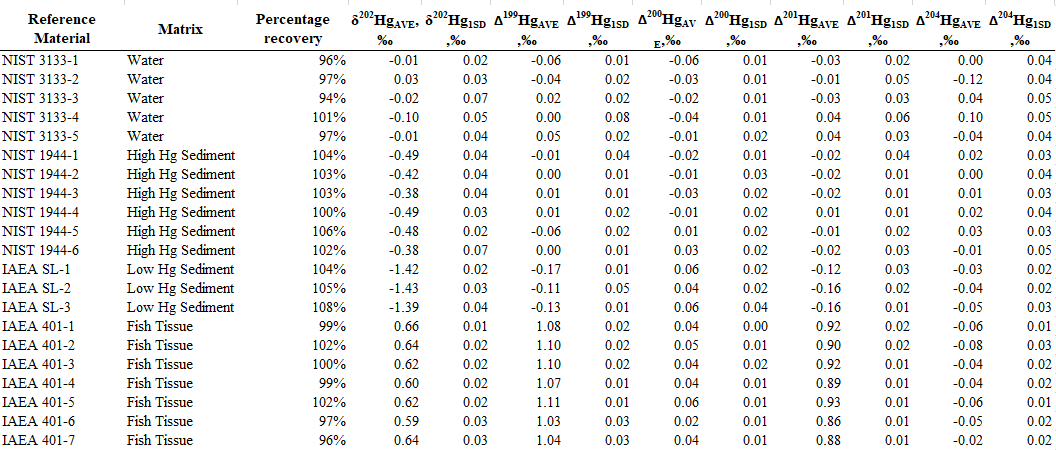


**Table S3**. Linear Combination fitting results for EXAFS analysis of soil samples.

| **Sample** | **Mercury Species** | | |
| --- | --- | --- | --- |
|  | **Cinnabar** | **Metacinnabar** | **Hg(Cyst)_2_** |
| Mine Tailings (New Furnace) | 100% | --- | --- |
| Mine Tailings (Old Furnace) | 48 ± 5% | 52 ± 7% | --- |
| Mine Waste (Unprocessed) | 79 ± 8% | --- | 21 ± 8% |

**References**

Bergquist BA, Blum JD. Mass-dependent and -independent fractionation of hg isotopes by photoreduction in aquatic systems. Science 2007; 318: 417-20.

Blum JD, Bergquist BA. Reporting of variations in the natural isotopic composition of mercury. Anal Bioanal Chem 2007; 388: 353-359.

Eckley CS, Luxton TP, McKernan JL, Goetz J, Goulet J. Influence of reservoir water level fluctuations on sediment methylmercury concentrations downstream of the historical Black Butte mercury mine, OR. Applied Geochemistry 2015; 61: 284-293.

Gehrke GE, Blum JD, Slotton DG, Greenfield BK. Mercury Isotopes Link Mercury in San Francisco Bay Forage Fish to Surface Sediments. Environmental Science & Technology 2011; 45: 1264-1270.

Janssen SE, Riva-Murray K, DeWild JF, Ogorek JM, Tate MT, Van Metre PC, et al. Chemical and Physical Controls on Mercury Source Signatures in Stream Fish from the Northeastern United States. Environmental Science & Technology 2019; 53: 10110-10119.

Sherman LS, Blum JD. Mercury stable isotopes in sediments and largemouth bass from Florida lakes, USA. Science of the Total Environment 2013; 448: 163-175.
